# Spontaneous adverse drug reactions reported in a thirteen-year pharmacovigilance program in a tertiary university hospital

**DOI:** 10.3389/fphar.2024.1427772

**Published:** 2024-12-05

**Authors:** E. Montané, Y. Sanz, S. Martin, C. Pérez-Mañá, E. Papaseit, O. Hladun, G. De la Rosa, M. Farré

**Affiliations:** ^1^ Service of Clinical Pharmacology, Hospital Universitari Germans Trias i Pujol, Barcelona, Spain; ^2^ Department of Pharmacology, Therapeutics and Toxicology, Universitat Autònoma de Barcelona, Barcelona, Spain

**Keywords:** pharmacovigilance, postmarketing drug safety, adverse drug reaction, spontaneous reporting systems, patient safety

## Abstract

**Objectives:**

We aimed to assess the characteristics of adverse drug reactions (ADRs) collected in a university hospital.

**Methods:**

A retrospective analysis of ADRs spontaneously reported in the Hospital Pharmacovigilance Program database (RutiRAM) over a 13-year period was conducted. The analysis included a description of ADRs [System Organ Class (SOC)] and their seriousness, the drugs involved [level 1 of the Anatomical Therapeutic Chemical (ATC) Classification System], drug-drug interactions, medication errors, drugs ‘under additional monitoring’, positive rechallenge, and the ‘pharmacovigilance interest’ of ADRs. An ADR was considered of ‘pharmacovigilance interest’ when it was serious, and/or produced sequelae, and/or affected the paediatric population, and/or when the suspected drug was ‘under additional monitoring’. Additionally, an exploratory analysis for bivariate associations through an automated method was performed.

**Results:**

A total of 2,148 spontaneous ADRs were registered in the RutiRAM database, with 92.5% recorded by medical doctors. The mean age of cases was 59.2 years (SD 20.9), range 1 day–99 years; 5.7% were paediatric, 46.2% adults, and 48.1% elderly. The drugs most often involved were anti-infectives (ATC group J), mainly amoxicillin-clavulanic acid. ‘Blood system disorders’ were the most frequent SOC ADRs, and skin rashes were the most frequent ADRs. The 63.2% of ADRs were considered of ‘pharmacovigilance interest’. Almost half of ADRs were hospital-acquired, and these were related to medication error; serious ADRs were related to drug-drug interactions and elderly patients, and involved drugs ‘under additional monitoring’ were related to younger ones.

**Conclusion:**

This is the first study to overview of ADRs reported in an HPVP over more than a decade. Almost two-thirds of the ADRs collected in the RutiRAM database are of sufficient quality to be classified as ‘pharmacovigilance interest’, and thus can contribute to signal detection and the issuing of drug alerts by pharmacovigilance systems. Analysing ADRs in hospitals contributes to patient safety by implementing relevant actions to prevent medication errors or ADRs, some of which can be applied to other centres.

## 1 Introduction

Adverse drug reactions (ADRs) are unwanted results of pharmacological therapy. ADRs worsen the quality of life of patients, increase hospital admissions, lengthen hospital stays, increase mortality, and represent a considerable economic burden for health systems (Pirmohamed et al., 2004; Montané and Castells, 2021; Sultana et al., 2013; Davies et al., 2009).

The definition of ADRs has evolved over time. The first and globally recognized definition was proposed by the World Health Organization (WHO) (World health organization technical report series, 1972); but in the last decade, a new European legislation on pharmacovigilance appeared, which is currently in force and has broadened the definition of ADR to ’A response to a medicinal product which is noxious and unintended‘, which includes off-label use, medication errors, drug abuse, and drug misuse (Commission directive 2010, 2010). The WHO has defined pharmacovigilance as ‘the science and activities relating to the detection, assessment, understanding, and prevention of adverse effects or any other medicine/vaccine related problems’ (World Health Organization, 2024).

The primary approach in pharmacovigilance to generate signals or alerts and to identify emerging safety concerns is through the spontaneous reporting of suspected ADRs. Its key benefits encompass its simplicity and cost-effectiveness, but the most acknowledged drawbacks involve underreporting and the inability to calculate incidence rates (Pal et al., 2013). Underreporting results in reduced method sensitivity, often leading to delays in signal detection (Hazell and Shakir, 2006). Priority ADR notifications include cases related to recently marketed medications that require further follow-up, events not previously documented, serious ADRs, and those that impact the paediatric patients (Guidelines for Detecting & Reporting Adverse Drug Reactions, 2014).

The major pharmacovigilance systems for the collection of spontaneous ADRs available worldwide are the Food and Drug Administration’s Adverse Event Reporting System (FAERS) (from the USA), European Medicines Agency’s (EMA) network system for reporting and evaluating suspected ADRs called Eudravigilance (from the European Union), the Japanese Adverse Drug Event Report (JADER) database, and the global database of individual case safety reports Vigibase (from the members of the WHO program for international drug monitoring) (The European network of centres for pharmacoepidemiology and pharmacovigilance guide on methodological standards in pharmacoepidemiology, 2024). The characteristics of the reported ADRs may vary in different countries and regions due to demographic and genetic characteristics of the population, and the patterns of drug consumption (Leporini et al., 2017). In Spain, the Spanish Pharmacovigilance System monitors medicine safety. Its main goal is to ensure medicines are safe and to identify, assess, and minimize potential risks. It operates under the European pharmacovigilance framework, coordinated by the Spanish Agency for Medicines and Medical Devices (AEMPS). There are 17 autonomous pharmacovigilance centres that contribute data to the national database (FEDRA), through spontaneous reports from physicians, pharmacists, nurses, and other healthcare providers, as well as patients and citizens. Hospitals report ADRs either individually or centrally through an Hospital Pharmacovigilance Program (HPVP) reporting their collected cases to the autonomous pharmacovigilance centre. Where they exist, the content of the HPVP may differ from one hospital to another, but they share the same objectives: detecting, quantifying, and preventing ADRs to increase patient safety.

Considering that the regulatory framework establishes pharmacovigilance as an activity of shared responsibility among all agents involved, such as hospitals, participation through their Pharmacovigilance Program is necessary for monitoring serious and even fatal ADRs, among others (Guideline on good pharmacovigilance practices GVP, 2017). Preliminary data from low-income countries have been published, but little is reported about data from tertiary care hospitals and their pharmacovigilance programs in high-income countries (Jha et al., 2009; Geer et al., 2016; Kaur et al., 2020; Lobo et al., 2013; Alsbou et al., 2015).

The aims of the present study were to determine the characteristics of ADRs in patients registered in the Hospital Pharmacovigilance Program database over a 13-year period and to assess their quality according to ‘pharmacovigilance interest’.

## 2 Methods

We followed the STROBE Statement to report the study sections and their contents (Vandenbroucke et al., 2007).

### 2.1 Study setting

The Germans Trias i Pujol Hospital is a tertiary care hospital with 734 beds for a population of about 850,000 people living in the Barcelonès Nord I Maresme area of Barcelona, in Catalonia, Spain.

The study was approved by the Research Ethics Committee of the Germans Trias i Pujol Hospital in 2019 and was conducted in 2023.

The HPVP at Germans Trias i Pujol Hospital was formally established in 2006, although some pharmacovigilance activity had been carried out previously. The main pharmacovigilance activities of this HPVP are to detect, quantify, and prevent ADRs as much as possible to increase inpatient safety. Currently, the detection of ADRs is powered by spontaneous notifications from healthcare professionals made through the hospital’s electronic yellow card. The registered ADRs come from any patient who is seen in the hospital, such as patients attending the emergency department, hospitalized patients, and outpatients followed up by hospital specialists. Suspected ADRs notifications, reported by different healthcare professionals, namely, medical doctors, nurses, medical students, and pharmacists of the hospital, are prospectively collected. A spontaneous report of an ADR includes the following minimal information: patient identification data, name of the suspected drug or drugs, description of the adverse reaction, and identification data of the reporter. Clinical pharmacologists are charged with collecting all the detailed data from electronic health record required for the yellow card. The Drug Safety Committee of the hospital accurately evaluates all the suspected ADRs. When the cases are considered possible, probable, or definite causality attribution according to the Spanish pharmacovigilance algorithm, the ADRs are included in the hospital registry named ‘RutiRAM database’. Incomplete ADR outcomes are annually updated until January 31 of the following year.

### 2.2 Study design

We conducted a retrospective analysis of all ADRs reported by health professionals at the Germans Trias i Pujol Hospital and registered in the RutiRAM database over 13 years, between 1 January 2010, and 31 December 2022.

### 2.3 Study population

All ADRs recorded in the RutiRAM database were selected and included in the study. The included cases were previously reported to the Spanish Pharmacovigilance System, except those in which the patient was included in a clinical trial, because these ADR reports follow a specific notification system.

### 2.4 Variables

The following information was extracted for each ADR from the registry: year reported, healthcare reporter, origin of the ADR, age and sex of the patient, type and system organ class related to the ADR, type and number of drugs involved, drugs ‘under additional monitoring’, drug-drug interaction, type of interaction, medication error, seriousness of the ADR, ‘pharmacovigilance interest’ of the ADR, positive rechallenge of involved suspected drug, and outcome of the ADR.

#### 2.4.1 Variable definitions and classifications

ADRs: descriptive terms of reactions were classified by the System Organ Classes (SOC) according to the MedDRA dictionary (Medical Dictionary for Regulatory Activities) (MedDRA Maintenance and Support Services Organization, 2023).

ADR origin: the origin of the ADRs was classified as hospital-acquired when the reaction occurred during hospitalization, in the emergency department, or in the area where patients receive treatments. ADRs that occurred in outpatient clinics or led to hospital admission were classified as non-hospital-acquired ADRs.

Age groups of the population: three groups were defined: paediatric (until 17 years old), adults (18–64 years), and elderly (65 years or more).

Drug classes: suspected drugs were classified according to the categories of the Anatomical Therapeutic Chemical (ATC) classification System (level 1) (WHO Collaborating Centre for Drug Statistics Methodology, 2024).

Nonprescription drugs: suspected nonprescription drugs were illegal drugs, herbal medicines, or dietary supplements, which are not classified in the ATC Classification System, therefore they were analysed separately.

Drug-drug interaction: if a drug-drug interaction was suspected a review of the literature was done to document the interaction. Drug-drug interactions were classified as either pharmacodynamic or pharmacokinetic. Pharmacodynamic interactions were defined as those in which drugs influence each other’s pharmacologic effect, and were evaluated if they were synergistic or antagonistic. Pharmacokinetic interactions were defined as those in which a drug could result in the increase or the decrease of plasma drug concentrations (Cascorbi, 2012).

Drugs ‘under additional monitoring’: drugs were classified as being or not ‘under additional monitoring’. ‘Additional monitoring’ is a term denoted by the EMA to medicines that are more intensively monitored than others (Medicines under additional monitoring, 2024). This is generally because there is less safety information available, for example, because the medicine has been recently marketed or there is limited data on its long-term use. A drug with additional follow-up is a drug that had an inverted black triangle (▼) on the package leaflet. A drug was denoted as ‘under additional monitoring’ if it was included in the EMA’s list of medicines ‘under additional monitoring’ according to the year in which the ADR occurred (List of medicines under additional, 2024).

Healthcare reporters: healthcare professionals were classified as doctors, pharmacists, nurses, and medical students. Nursing assistants and radiology technicians were included in the nurses group. Medical students from the Germans Trias i Pujol Teaching Unit (Autonomous University of Barcelona) do their internships at the hospital, and during the fifth year they have a voluntary learning activity that consists of identifying and collecting suspected ADRs.

Medication error: a medication error was defined according to the National Coordinating Council for Medication Error Reporting and Prevention: ‘medication error is any preventable event that may cause or lead to inappropriate medication use or patient harm while the medication is in the control of the healthcare professional, patient, or consumer. Such events may be related to professional practice, healthcare products, procedures, and systems, including prescribing, order communication, product labelling, packaging, and nomenclature, compounding, dispensing, distribution, administration, education, monitoring, and use (About Medication Errors, 2024).

‘Pharmacovigilance interest’: an ADR was considered of pharmacovigilance interest when it was serious, and/or produced sequelae, and/or affected the paediatric population, and/or when the suspected drug was ‘under additional monitoring’ (Montané and Santesmases, 2020).

Rechallenge: a positive rechallenge was considered when following an adverse reaction that had been resolved by withdrawal of the suspected drug, the drug was re-administered and the same ADR reappeared (Stephens, 1983; Girard, 1987).

Seriousness of ADRs: a serious ADR was defined according to the International Conference on Harmonization (ICH) guideline E2D which encompasses ADRs that are fatal, life-threatening, requiring hospital admission or prolongation of hospital stay, causing persistent or significant disability/incapacity, congenital anomaly/congenital defect or medically important. The remaining cases were defined as non-serious ADRs (European Medicines Agency, 2004).

Time periods: ADRs were grouped in two periods, the first from 2010 to 2016, and the second from 2017 to 2022.

### 2.5 ADR causality assessment

The Drug Safety Committee of the Hospital was composed of clinical pharmacologists, one of them being a senior specialist in pharmacovigilance, and specialised nurses. It was responsible for assessing the causality attribution of all reported ADRs in the HPVP. Each reported case was evaluated in detail by clinical pharmacologists using the modified Karch and Lasagna algorithm, that is used by the Spanish Pharmacovigilance System (Aguirre and García, 2016). This algorithm assesses the following five items: temporal relationship between the onset of the drug and onset of the reaction, knowledge of the reaction in the literature, the clinical effect of withdrawal and rechallenge to the drug involved, assessment of alternative causes, and background clinical factors that may have contributed to the onset of the reaction (Aguirre and García, 2016). ADRs have been included in the RutiRAM database if the Drug Safety Committee scored their causality attribution as ‘possible’, ‘probable’, or ‘definite’.

### 2.6 Statistical analysis

For descriptive analysis, we used the number of cases and percentages for categorical variables; median and range for ordinal variables; and mean and standard deviation (SD) for continuous variables. ADRs by SOC and involved drugs by ATC were compared between two periods (2010–2016 vs. 2017–2022) using Chi-square or the Exact Fisher Test. Characteristics of serious and non-serious ADRs were compared using Chi-square Test.

Statistical analysis was performed using the SPSS statistical software package for Windows, version 29.0 (SPSS Inc., Chicago, IL, USA).

#### 2.6.1 Exploratory data analysis

A thorough automated method for exploratory data analysis (AutoDiscovery, Butler Scientifics, Barcelona, Spain) was conducted to evaluate bivariate associations with the ADR origin, ADR seriousness and drugs ‘under additional monitoring’. The suitable statistical approach was chosen based on the type of data and the distribution of the variables in each case, as assessed by AutoDiscovery. The statistical methods utilized were:

a) Spearman’s Rank Correlation: for numerical variable pairs.
b) Variance Analysis: for categorical (factor) and numerical (response) variable pairs, specifically: ANOVA one-way: when the response fits the normal distribution (D'Agostino/Pearson test); U Mann-Whitney: when the response does not fit the normal distribution and the factor has exactly two categories; and Kruskal–Wallis: when the response does not fit the normal distribution and the factor has more than two categories.
c) Cramer’s V Contingency Index: for categorical variable pairs.

This procedure was implemented in each potential subgroup of the dataset, created based on previously selected stratification factors (demographics, characteristics of the ADR and features of the drugs). Subgroups or associations having a sample size of fewer than 5, a sample size that is less than 1% of the total, or a significance level α (two-sided test) of 0.05 or higher were automatically discarded.

Due to the nature of this multi-test approach, a False Discovery Rate (FDR) correction method (Benjamini–Hochberg, 5% false discovery rate) was applied, providing a new *p*-value threshold of 0.0004 for highly significant results.

Lastly, expert evaluation of the recorded findings, particularly highly significant results, was undertaken to identify the most pertinent outcomes related to the initial objectives.

## 3 Results

During the 13-year study period, a total of 2,148 spontaneous ADRs cases were recorded in the RutiRAM database. The number of ADRs recorded annually ranged from 79 to 230, with an average of 165 ADRs per year. Of the ADRs, 92.5% (1,987/2,148) were reported by medical doctors, 3.1% (67/2,148) by nurses, 2.6% (55/2,148) by pharmacists, and 1.8% (39/2,148) by medical students.

These 2,148 suspected ADRs occurred in 1,905 patients (198 patients had two ADRs each, 35 patients presented three ADRs, eight patients had four ADRs, one patient had five ADRs, and another six ADRs). The mean age of cases was 59.2 years (SD 20.9), ranging from 1 day to 99 years (median 63 years), of which 53.3% (1,145/2,148 cases) were males. The distribution of ADRs by age group was: 5.7% were paediatric (122/2,148), 46.2% were adults (993/2,148), and 48.1% were elderly (1,033/2,148).

In 1.8% of ADRs (38/2,148 cases), they occurred in patients included in clinical trials.

### 3.1 Characteristics of ADRs

The most frequent ADR classified by SOC were blood and lymphatic system disorders (18.4%, 394/2,148 cases) and immune system disorders (14.1%, 302/2,148 cases) (Table 1). Generalized skin rash or erythema was the most frequent ADR (11.5%, 247/2,148 cases). The remaining most frequent types of ADRs are detailed in Table 2.

**TABLE 1:** Distribution of adverse drug reactions (ADRs) by organ and systems classification (SOC).

Organ and systems classification (SOC)^a^	N (%)
Blood and lymphatic system disorders	394 (18.4)
Cardiac disorders	87 (4.0)
Congenital, familial and genetic disorders	6 (0.3)
Ear and labyrinth disorders	3 (0.1)
Endocrine disorders	67 (3.1)
Eye disorders	8 (0.4)
Gastrointestinal disorders	66 (3.1)
General disorders and alterations at site of administration	10 (0.4)
Hepatobiliary disorders	201 (9.4)
Immune system disorders	302 (14.1)
Infections and infestations	229 (10.7)
Traumatic injuries, intoxications and complications of therapeutic procedures	44 (2.0)
Metabolism and nutrition disorders	79 (3.7)
Musculoskeletal disorders	47 (2.2)
Neoplasm benign, malignant and unspecified	10 (0.5)
Nervous system disorders	145 (6.8)
Pregnancy, puerperium and perinatal conditions	2 (0.1)
Psychiatric disorders	18 (0.8)
Renal and urinary disorders	64 (3.0)
Reproductive system and breast disorders	5 (0.2)
Respiratory, thoracic and mediastinal disorders	50 (2.3)
Skin and subcutaneous tissue disorders	287 (13.3)
Vascular disorders	24 (1.1)
Total	2,148 (100)

^a^ Medical Dictionary for Regulatory Activities (MedDRA^®^).

**TABLE 2:** Distribution of the most frequent Adverse Drug Reactions (ADRs) type reported and their most frequent involved drugs.

ADR^a^ and most frequent involved drugs	Number of ADRs	%
Generalized erythema Antibiotics/metamizole/iodinate contrasts	247	11.5
Acute hepatitis Antibiotics (mainly amoxicillin-clavulanic acid)/statins (mainly atorvastatin)	97	4.5
Cerebral haemorrhage Acenocoumarol and/or AAS	87	4.1
Cholestasis Antibiotics (mainly amoxicillin-clavulanic acid)	39	1.8
Elevation of liver function tests Antibiotics/statins (mainly atorvastatin)	38	1.7
Agranulocytosis Beta-lactam antibiotics and/or metamizole	51	2.4
Renal failure Vancomycin/NSAID	48	2.2
Pseudomembranous colitis Beta-lactam antibiotics	46	2.1
Pneumonia Monoclonal antibodies and corticosteroids	46	2.1
Thrombocytopenia Enoxaparin	39	1.8
Angioedema Miscellaneous	38	1.8
Leukopenia Beta-lactam antibiotics	31	1.4
Infusion reaction Monoclonal antibodies/amphotericin B	29	1.3
Anaphylaxis Beta-lactam antibiotics/metamizole	28	1.3
DRESS syndrome Allopurinol/antibiotics/metamizole	27	1.3
Hematoma soft parts Enoxaparin	27	1.3
Localized erythema Antibiotics (mainly ciprofloxacin)	26	1.2
Anaphylactic shock Metamizole/beta-lactam antibiotics	26	1.2
Pancytopenia Antibiotics/antineoplastics	26	1.2
Hypophosphatemia Iron carboxymaltose	26	1.2
Pneumonitis Monoclonal antibodies (mainly rituximab)	25	1.2
SIADH syndrome (inadequate secretion of ADH syndrome) Selective serotonin reuptake inhibitors and diuretics	20	0.9
Total ADRs	2,148	100

^a^ ADRs, reported for more than 19 cases.

Types of ADRs, reported for more than four cases (number of ADRs): eosinophilia (18), drowsiness (17), long QT, syndrome (16), septic shock (15), pancreatitis (15), hypersensitivity reaction (14), bronchospasm (14), generalized erythema (14), encephalopathy (14), hyponatremia (13), flu infection (13), upper gastrointestinal bleeding (12), haematuria (10), hypopotassaemia (10), digoxin poisoning (10), hypoglycaemia (9), convulsion (9), acute generalized exanthematic pustulosis (9), diarrhoea (8), atrioventricular block (8), cytomegalovirus reactivation (7), rectorrhagia (7), hyperpotassaemia (7), internal hematoma (7), herpes zoster infection (6), malignant neuroleptic syndrome (6), symmetrical drug-related intertriginous and flexural exanthema (SDRIFE) (6), lactic acidosis (6), red man syndrome (6), rhabdomyolysis (5), autoimmune hepatitis (5), toxic epidermal necrolysis (5), infusion lumbar pain (5), hepatic failure (5), leukocytoclastic vasculitis (5), erythema multiforme (5), and hyperglycaemia (5).

Of the total, 55.8% (1,198/2,148 cases) of ADRs were serious, 171 of which were fatal ADRs (8%, 171/2,148). A total of 47.5% (1,020/2,148 cases) of reported ADRs were hospital-acquired. A total of 1,358 ADRs (63.2%, 1,358/2,148) were considered ADRs of quality.

**FIGURE 1:**
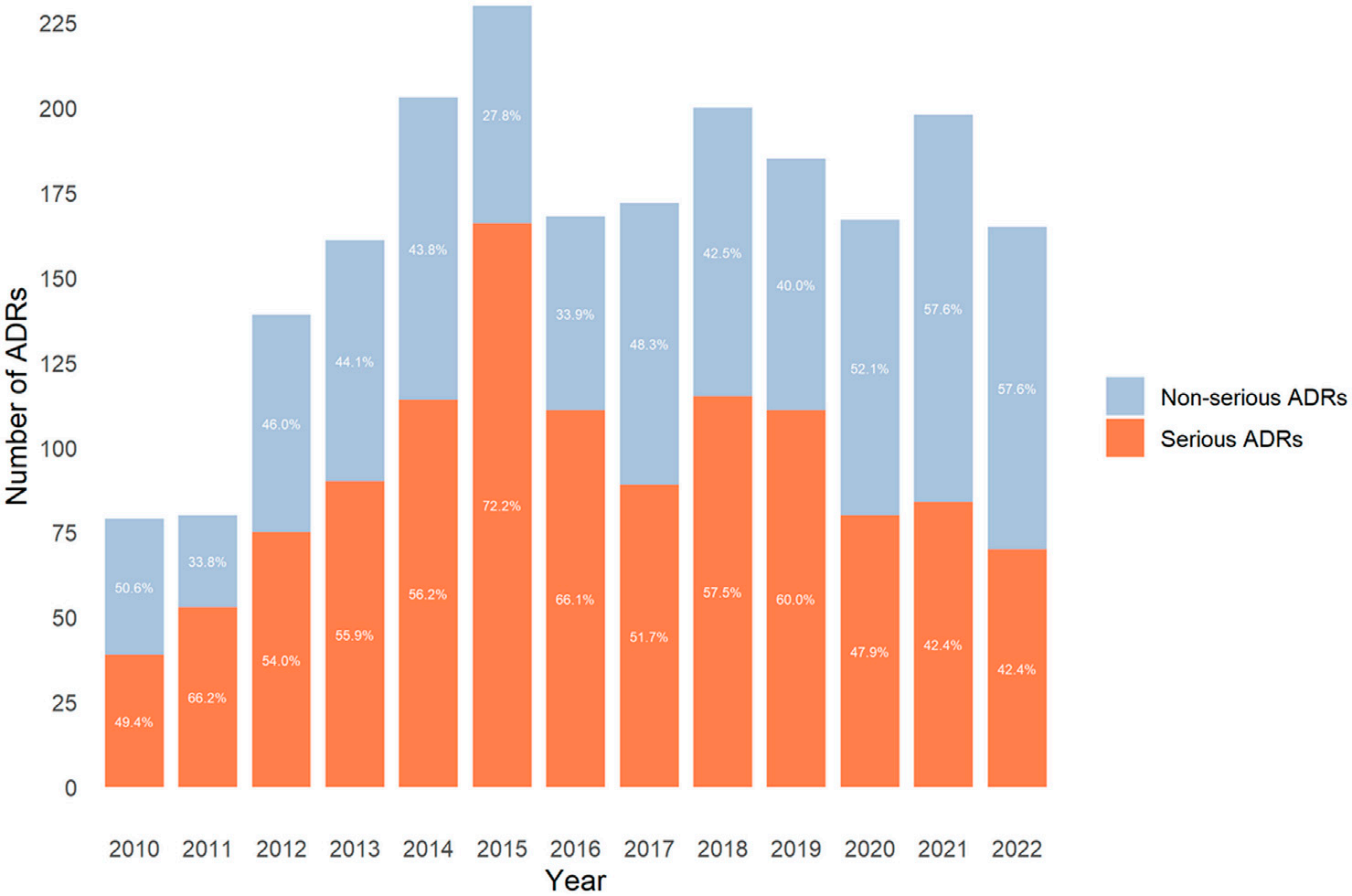
Distribution of the number of reported adverse drug reactions (ADRs) and seriousness by year.

The evolution percentage of serious ADRs over time showed values of around 50% (ranging from 42% to 60%) except in 2011, 2015, and 2016, which were >60% (Figure 1).

### 3.2 Characteristics of suspected drugs

The median number of suspected drugs was 1.0 (ranging from 1 to 10). In 33.5% of ADRs (719/2,148 cases) there was more than one suspected drug involved: two drugs in 473 ADRs (22%, 473/2,148), three in 173 ADRs (8.1%, 173/2,148), four in 46 ADRs (2.1%, 46/2,148), and five or more in 27 ADRs (1.3%, 27/2,148). In 18.3% of ADRs (393/2,148) a drug-drug interaction was considered the cause of ADR; 93.9% of these were pharmacodynamic (369/393) and 6.9% (27/393) were pharmacokinetic interactions. The ADRs caused by pharmacodynamic interactions were mainly infections (38.2%, 141/369) related to antineoplastics and/or immunosuppressants drugs. In 240 ADRs (11.2%, 240/2,148) a drug ‘under additional monitoring’ was involved. In 134 ADRs (6.2%, 134/2,148) a positive rechallenge with the suspected drug was reported. Medication errors were observed in 4.4% of ADRs (95/2,148 cases).

In total, there were 3,170 suspected drugs involved in 2,148 ADRs; 27.2% of involved drugs (863/3,170) were classified in the ATC category J (Anti-infectives for systemic use) and 20.9% (663/3,170) in the category L (Antineoplastic agents and immunomodulators) (Table 3). There were 514 different involved drugs, being the most frequently reported amoxicillin-clavulanic acid (5.3%, 114/2,148 cases) and metamizole (4.9%, 105/2,148 cases) (Table 4). In 13 patients (12.4%, 13/105), metamizole was suspected of causing agranulocytosis or neutropenia, and in four of these reports it was the suspected drug concomitantly with beta-lactam antibiotics. Nonprescription drugs, including herbal medicines, dietary supplements, or illegal drugs were implicated in 20 ADRs (0.93%, 20/2,148). (Table 5).

**TABLE 3:** Distribution of the Anatomical Therapeutic Chemical (ATC) classification system of involved drugs.

ATC category	Therapeutic area	N	%
A	Alimentary tract and metabolism	132	4.2
B	Blood and blood forming organs	350	11.0
C	Cardiovascular system	316	10.0
D	Dermatological	11	0.3
G	Genito-urinary system and sex hormones	10	0.3
H	Systemic hormonal preparations, excluding sex-hormones and insulins	143	4.5
J	Anti-infectives for systemic use	863	27.2
L	Antineoplastic and immunomodulating agents	663	20.9
M	Musculo-skeletal system	126	4.0
N	Nervous system	447	14.1
P	Antiparasitic products, insecticides and repellents	17	0.5
R	Respiratory system	34	1.1
S	Sensory organs	2	0.1
V	Various	56	1.8
	Total	3,170	100

**TABLE 4:** The most frequent involved drugs in adverse drug reactions (ADRs).

Drugs^a^	Number of ADRs	%
Amoxicillin-clavulanic acid	114	5.3
Metamizole	105	4.9
Acenocoumarol	78	3.6
Acetylsalicylic acid	76	3.5
Enoxaparin	73	3.4
Prednisone	68	3.2
Vancomycin	55	2.6
Ceftriaxone	52	2.4
Levofloxacin	49	2.3
Omeprazole	47	2.2
Methotrexate	43	2.0
Piperacillin-tazobactam	43	2.0
Ciprofloxacin	41	1.9
Meropenem	39	1.8
Methylprednisolone	36	1.7
COVID-19 vaccine	36	1.7
Rituximab	36	1.7
Furosemide	34	1.6
Dexketoprofen	33	1.5
Infliximab	31	1.4
Mycophenolate acid	30	1.4
Total ADRs	2,148	100

^a^ Drugs involved in at least 30 ADRs.

Drugs involved in more than four cases (number of ADRs): Cotrimoxazole (29), iron carboxymaltose (29), amiodarone (28), clopidogrel (28), tacrolimus (28), cefepime (26), enalapril (26), iomeprol (26), atorvastatin (25), ibuprofen (25), clindamycin (22), hydrochlorothiazide (22), cyclosporine (20), spironolactone (20), allopurinol (19), dexamethasone (19), sodium heparin (19), azathioprine (18), cyclophosphamide (18), nivolumab (18), cloxacillin (17), fentanyl (17), simvastatin (17), quetiapine (16), rifampicin (16), adalimumab (15), morphine (15), paracetamol (14), tocilizumab (14), tramadol (14), ceftazidime (13), cefuroxime (13), linezolid (13), paclitaxel (13), cefuroxime (12), cytarabine (12), digoxin (12), isoniazid (12), leflunomide (12), metformin (12), amikacin (11), phenytoin (11), propofol (11), sertraline (11), voriconazole (11), beta-lactams (10), cisplatin (10), dabigatran (10), diuretic (10), docetaxel (10), doxorubicin (10), fingolimod (10), gabapentin (10), gentamicin (10), olanzapine (10), ondansetron (10), pembrolizumab (10), rocuronium (10), valproic acid (10), azithromycin (9), diltiazem (9), haloperidol (9), lorazepam (9), mirtazapine (9), natalizumab (9), oxcarbazepine (9), vincristine (9), apixaban (8), cefotaxime (8), diclofenac (8), hydroxychloroquine (8), immunoglobulin (8), liposomal amphotericin B (8), metronidazole (8), risperidone (8), salbutamol (8), amoxicillin (7), bisoprolol (7), bortezomib (7), capecitabine (7), cetuximab (7), citalopram (7), everolimus (7), iodixanol (7), metoclopramide (7), remdesivir (7), torasemide (7), zoledronic acid (7), alprostadil (6), bictegravir/emtricitabine/tenofovir alafenamide (6), clonazepam (6), doxycycline (6), etanercept (6), fluoxetine (6), foscarnet (6), hydralazine (6), ionidated contrast agent (6), isotretinoin (6), lenalidomide (6), levetiracetam (6), lidocaine (6), losartan (6), sunitinib (6), alteplase rtpa (5), bendamustine (5), clomethiazole (5), durvalumab (5), etoposide (5), fluconazole (5), imatinib (5), itraconazole (5), lamivudine (5), naproxen (5), paroxetine (5), pemetrexed (5), ribavirin (5), ritonavir (5), trazodone (5), triamcinolone (5), valsartan (5).

**TABLE 5:** Characteristics of adverse drug reactions (ADRs) involving herbal medicines, dietary supplements or illegal drugs.

	Product	ADR	Concomitant suspected drug	Seriousness
Herbal medicines	Aloe vera	Acute hepatitis	Interferon beta	Serious
	Colloidal gold	Acute hepatitis	None	Serious
	Copalchi	Cholestasis	None	Non serious
	Fucus + copalchi	Acute hepatitis	None	Serious
	Goji berries	Hyperpotassaemia	Enalapril	Serious
	Hedera	Tachycardia and urticaria	Ibuprofen	Serious
	Melissa officinalis	Somnolence	None	Serious
	Matcha green tea	Acute hepatitis	None	Serious
	Red yeast rice	Autoimmune hepatitis	None	Serious
Dietary supplements	Chlorine dioxide	Haemolytic anaemia	None	Serious
	Collagen + magnesium	Rhabdomyolysis and hepatitis	None	Serious
	Herbalife	Cholestasis	Atorvastatin	Non serious
	Oxid nitric	Myalgia	None	Serious
	Spascupreel	sudden death	None	Serious (death)
	Valentus Slimroast Optimum	Agranulocytosis	Naproxen/hydrochlorothiazide	Serious
	X-treme	Arthralgia	None	Serious
Illegal drugs	Cocaine	Vasculitis	None	Non serious
	Cocaine	Agranulocytosis	None	Serious
	Cocaine	Thrombotic microangiopathy	Ciprofloxacin/ustekinumab	Serious
	Cocaïne + heroin + amphetamine	Acute hepatitis	Paracetamol	Serious

### 3.3 ADR outcome

The 81.8% of ADRs (1,758/2,148 cases) fully recovered, 2.7% of ADRs (57/2,148) had some sequelae, and in 11.1% of ADRs the patient died (239/2,148), of which 8% were drug-related death (DRD) (171/2,148) (in the remaining patients (68/2,148) the ADR was not the cause of the death). Additionally the 2.7% of ADRs (57/2,148) were ongoing and in 1.7% (37/2,148) the outcome was unknown when the data were extracted.

### 3.4 Comparison of the characteristics of ADRs according to time periods

The number of ADRs reported during the 2010–2016 period was similar to those reported during the 2017–2022 period (1,060, 49.3% vs. 1,088, 50.7%; *p* = 0.5600).

**FIGURE 2:**
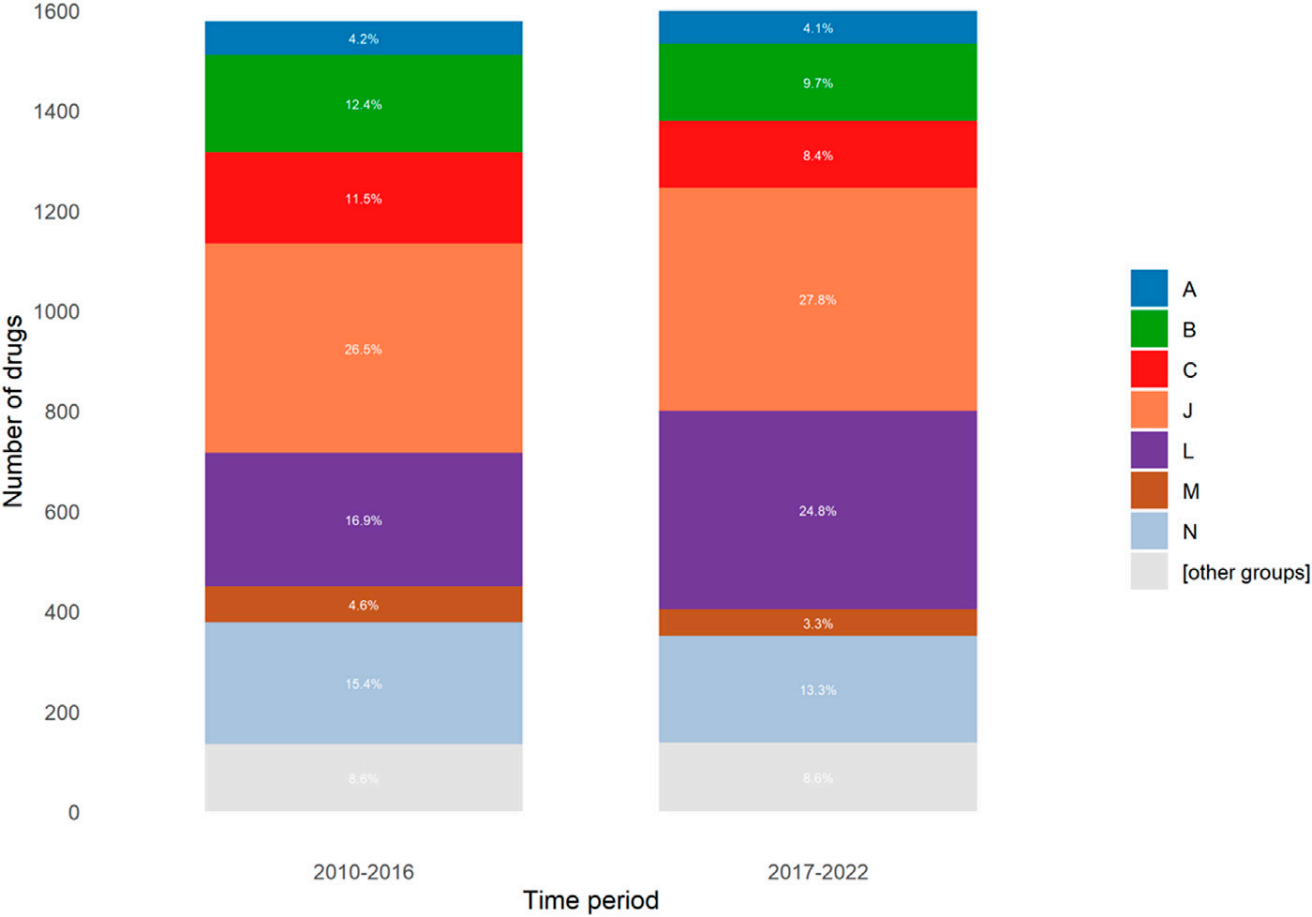
Distribution of involved drugs according to ATC classification for each period.

The ATC of the drugs involved in ADRs was similar between the two periods, except for category L drugs, which were higher in the 2017–2022 period (16.9% vs. 24.8%; *p* < 0.0001) and category C drugs, which were lower in the second period (11.5% vs 8.4%; *p* = 0.003) (Figure 2).

**FIGURE 3:**
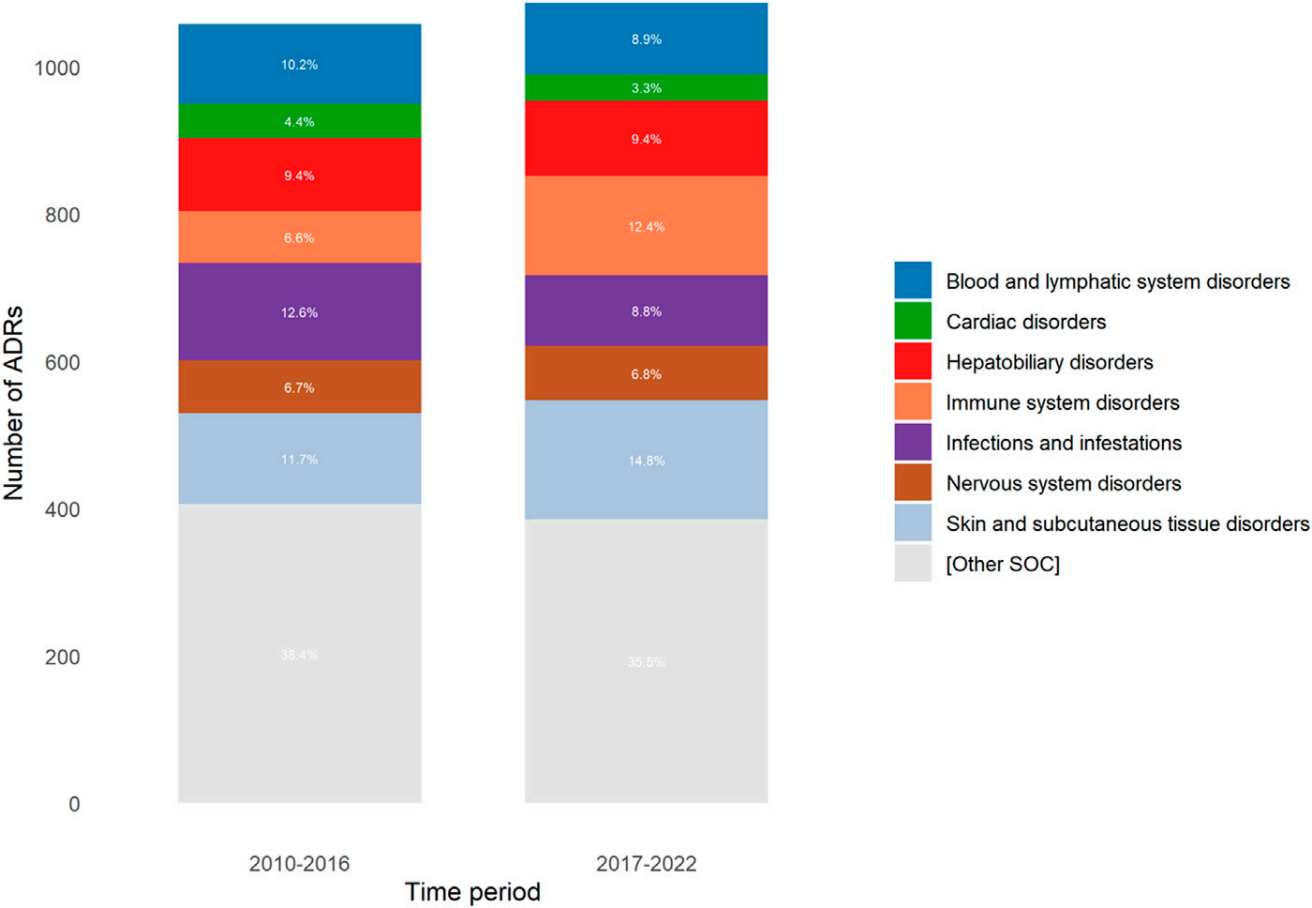
Distribution of adverse drug reactions (ADRs) according to SOC (System Organ Classification) for each period.

The SOC of reported ADRs was similar between the two periods, except for those corresponding to ‘Immune system disorders’ and ‘Skin and subcutaneous tissue disorders’, which were higher in the 2017–2022 period (6.6% vs.12.4%; *p* < 0.0001, and 11.7% vs.14.8%; *p* = 0.0359; respectively), and for those corresponding to ‘Infections and infestations’ and ‘Blood and lymphatic system disorders’, which were lower in the 2017–2022 period (12.6% vs.8.8%; *p* = 0.0052, and 10.2% vs. 8.9%, *p* = 0.0001; respectively) (Figure 3).

### 3.5 Comparison of ADRs characteristics according to seriousness

**FIGURE 4:**
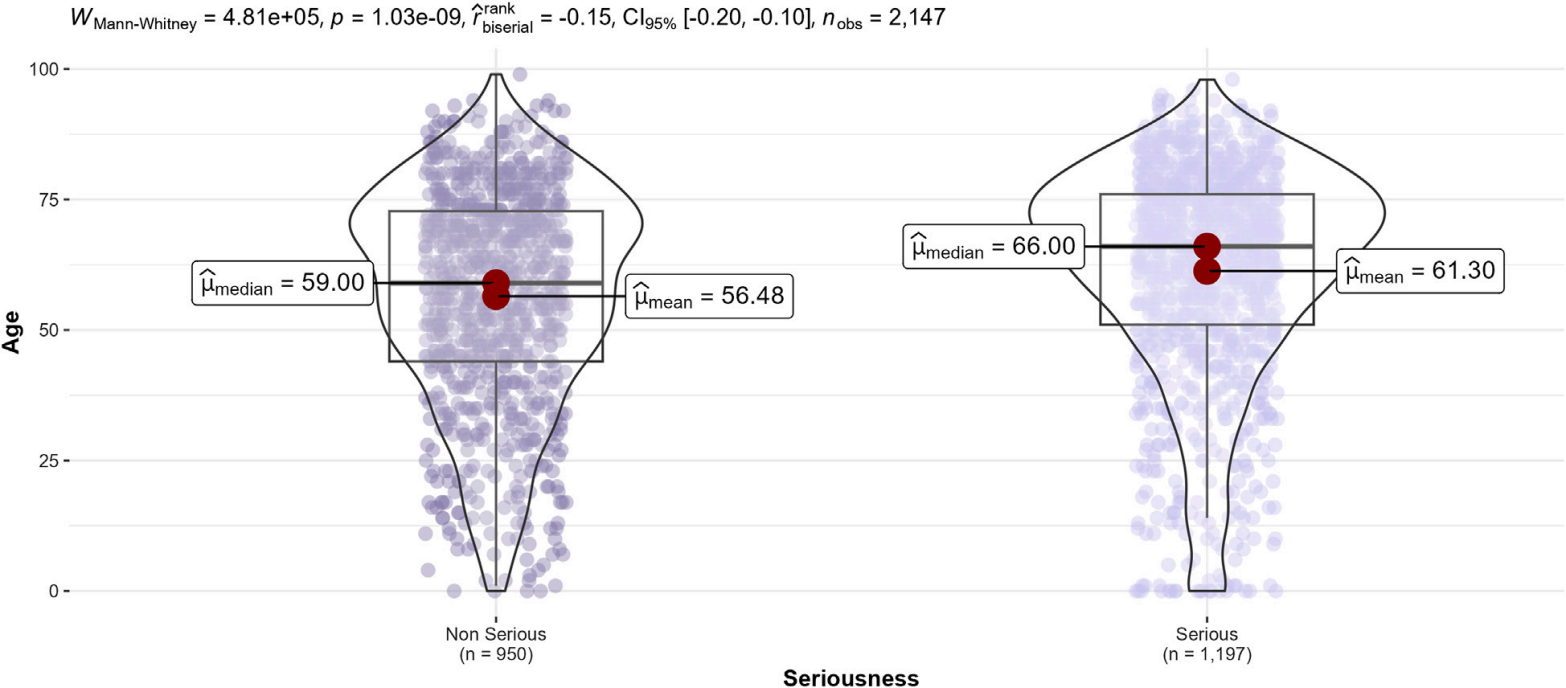
Distribution of patients’ age of adverse drug reactions according to seriousness.

Comparing of ADRs characteristics according to seriousness of ADRs showed that serious ADRs occurred in older patients (median age, 66 vs. 59 years, *p* < 0.0004) (Figure 4), drug-drug interactions were more frequently implicated (25.7% vs. 8.9%, *p* < 0.0002), as well as medication errors (4.9% vs. 3.7%, *p* < 0.001). On the other hand, serious ADRs were less often hospital-acquired (36.7% vs. 61.1%, *p* < 0.001), had fewer positive rechallenges (4.3% vs. 8.5%, *p* < 0.0002) and involved drugs ‘under additional monitoring’ less frequently (8.5% vs. 9.7%, *p* = 0.002) (Table 6).

**TABLE 6:** Comparison of characteristics of ADRs according to seriousness.

	Serious ADRs N = 1,198	Non-serious ADRs N = 950	Total N = 2,148	P
Sex, men (n, %)	619 (51.7%)	526 (55.4%)	1,145 (53.3%)	0.088
Age (years), median (range)	66 (0–98)	59 (0–99)	63 (0–99)	0.004
Hospital-acquired ADR	440 (36.7%)	580 (61.1%)	1,020 (47.5%)	<0.001
Drug-drug interaction	308 (25.7%)	85 (8.9%)	393 (18.3%)	<0.001
Drugs ‘under additional monitoring’	102 (8.5%)	92 (9.7%)	194 (9%)	0.002
Positive rechallenge	53 (4.3%)	81 (8.5%)	134 (6.2%)	<0.001
Medication error	59 (4.9%)	35 (3.7%)	94 (4.4%)	<0.001

### 3.6 Exploratory data analysis

Comparisons according to the origin of ADRs showed that hospital-acquired ADRs were more frequently related to error medication (6.5% vs. 2.4%, *p* < 0.0002) and to serious ADRs (67.2% vs. 43.1%, *p* < 0.0002). On the other hand, hospital-acquired ADRs were less related to drug-drug interactions (13.8% vs. 22.3%, *p* < 0.0002) and to drugs ‘under additional monitoring’ (3.6% vs. 13.9%, *p* < 0.0002), and less produced infections SOC (5.1% vs. 15.6%, *p* < 0.0002).

**FIGURE 5:**
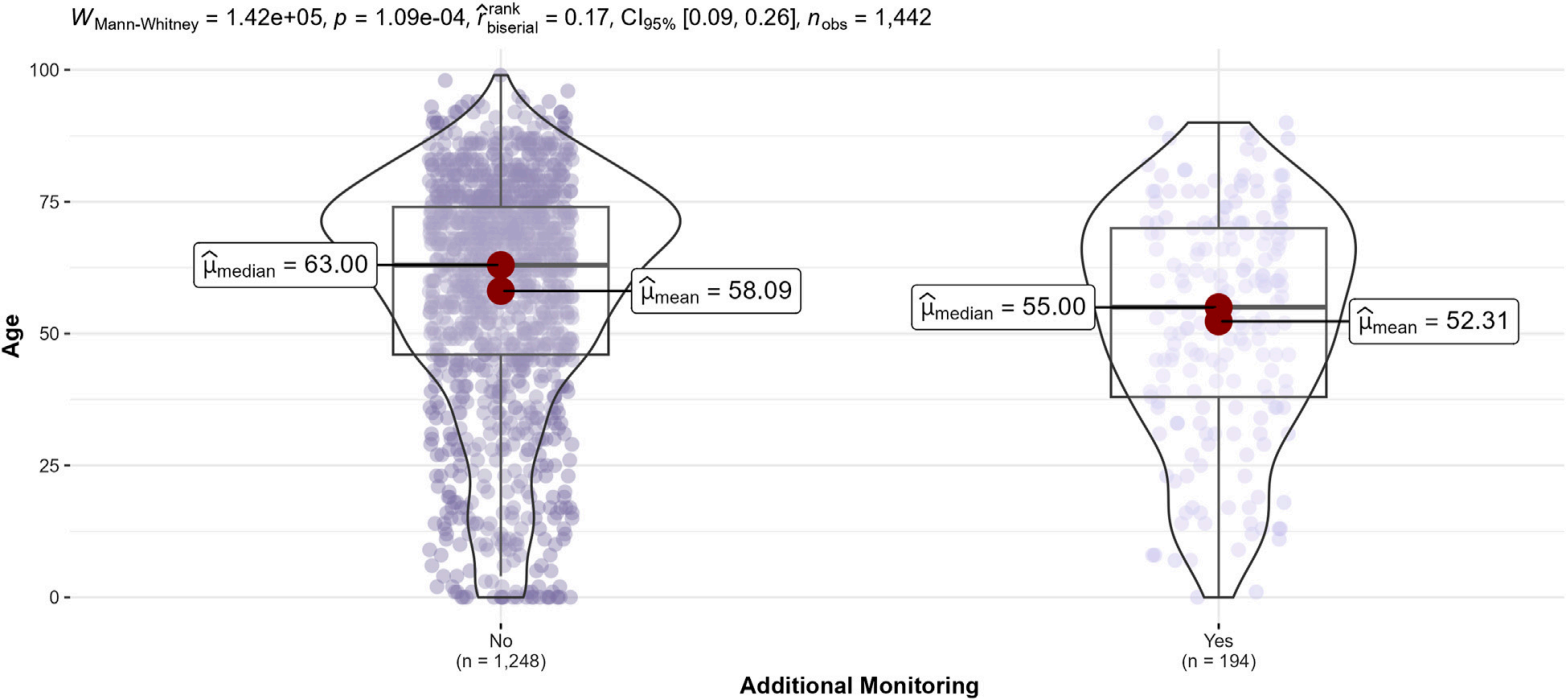
Distribution of patients’ age of adverse drug reactions according to drugs ‘under additional monitoring’ involved.

Comparisons according to ADRs with drugs ‘under additional monitoring’ involved (Figure 5) showed that patients with these ADRs were younger (median age 55 vs. 63 years, *p* < 0.0004). On the other hand, ADRs with a drug ‘under additional monitoring’ were less frequently related to the immune system disorder SOC (5.7% vs. 11.3%, *p* < 0.0004).

## 4 Discussion

Some medical institutions have developed ADR and medication error surveillance systems that are part of the HPVP, integrating pharmacovigilance into clinical practice and collaborating with the national pharmacovigilance system. In general, data on hospitals reporting ADRs to Pharmacovigilance Systems are scarce (Hazell and Shakir, 2006). In our centre, the number of suspected cases of ADR detected by HPVP is far from what would be expected based on the estimated incidences of ADR in the hospital setting (European Comission, 2024). There is clear underreporting as only a small proportion of the ADRs that occur are reported. According to the incidence described in the literature and considering that about 20,000 patients are admitted to the hospital each year, it is expected that approximately 1,000 patients will be admitted for an ADR and 1,000 patients will present an ADR during their hospital stay per year. Given that the RutiRAM database recorded an average of 165 ADR notifications per year, which represents 8.2% of the expected ADRs per year, there is a clear underreporting of ADRs; nevertheless, these results are similar to those described in other studies (Hazell and Shakir, 2006). On the other hand, healthcare professionals regularly report suspected ADRs to the HPVP as a sign of their commitment to pharmacovigilance. Regarding the profile of these healthcare professionals, in our study the reports were mostly submitted by physicians; in contrast to other pharmacovigilance programs wherereports were mostly made by pharmacists or nurses (Pérez-Ricart et al., 2019; Molina-Castiella and Napal-Lecumberri, 1999; Abu Esba et al., 2021). This could be explained by the fact that the HPVP in our hospital is designed and implemented by physicians who are specialised in clinical pharmacology. In any case, the most relevant aspect is that the participation of different categories of health professionals enriches the Pharmacovigilance Program because each group will observe different kinds of drug related problems (The importance of pharmacovigilance, 2002). Unlike other pharmacovigilance systems, patients did not participate in the reporting of ADRs in our HPVP.

The age of patients reported in our study and the slightly high prevalence of males were similar to those in another study (Pérez-Ricart et al., 2019; Brodsky et al., 2014). The most frequent ADR, as classified by SOC, was Blood and lymphatic system disorders and Immune system disorders, differing from data in other pharmacovigilance studies (Aagaard et al., 2012); where Skin and subcutaneous tissue disorders are globally the most frequently ADR reported, probably due to the fact that these ADRs come from primary healthcare settings, which are included in pharmacovigilance systems (Leporini et al., 2017; Brodsky et al., 2014; Marques et al., 2014), or due to their easier recognition by healthcare professionals (Pérez-Ricart et al., 2019).

On the other hand, ‘Antibacterials for systemic use’ (ATC code J) and/or ‘Antineoplastic agents’ (ATC code L) were the therapeutic subgroups mainly implicated in our study and several pharmacovigilance studies (Leporini et al., 2017; Pérez-Ricart et al., 2019; Molina-Castiella and Napal-Lecumberri, 1999; Brodsky et al., 2014). In contrast, these data differ from a previous study conducted in our hospital focusing on drug-related deaths, where the most frequently implicated therapeutic drugs were ‘Antineoplastic agents’ (ATC code L) and those of ‘Blood and blood forming organs’ (ATC code B) (Montané et al., 2018). The most frequently involved drugs in ADRs were amoxicillin-clavulanic acid and metamizole, both highly consumed in our country in primary care and in hospitalized patients. None of them are considered critical drugs by the EMA (The first Union list of, 2023); thus, they do not require any special supervision, unlike anticoagulants, for example, that must be carefully selected, monitored, and evaluated (O'Donnell, 2012). However, it is important to mention that recently, the EMA conducted a reassessment of the safety of metamizole due to cases of agranulocytosis and established risk minimization measures (EMA recommends measures to minimise, 2024). Thus, we would like to point out that metamizole was involved in 13 cases of agranulocytosis.

More than half of the reported ADRs were serious, and specifically 8% were DRD. These proportions are higher than in other studies probably due to reporting bias, since in our hospital’s HPVP we encourage reporting DRDs because it is a topic of interest to us and we have published results from previous studies (Montané et al., 2018; Arellano et al., 2021). In addition, in studies of pharmacovigilance system data, the ADRs registered are generally milder than those reported in hospitals, since primary healthcare is usually the main reporting institution (Molina-Castiella and Napal-Lecumberri, 1999).

Almost two-thirds of the ADRs were considered of ‘pharmacovigilance interest’ because they met the priority reporting criteria of pharmacovigilance systems (Guidelines for Detecting & Reporting Adverse Drug Reactions, 2014); this could be considered as an indicator of high-quality of the ADRs registered in RutiRAM database. In support of this idea, we have identified that the paediatric cases included in RutiRAM generated drug safety alerts from the Spanish Medicines Agency as explained in a previous published article (López-Valverde et al., 2021); therefore, we stress the importance of reporting and recording ADRs that occur in the hospital setting, thus contributing to the generation of drug safety alerts at national or international level (Filippi-Arriaga et al., 2023). Furthermore, despite the major limitations of the spontaneous ADR reporting systems, HPVP role is valuable in monitoring internal patterns and carrying actions for improving patient safety, which can be addressed through internal hospital policies (Abu Esba et al., 2021). Examples of actions carried out in our centre are the warning of hypophosphatemia in the electronic application when prescribing intravenous iron in hospitalised patients and the mandatory reporting of drug allergies in the electronic prescription application to avoid prescription errors. To provide feed-back to ADRs reporters, annual sessions are held to review trends and summarize the ADR analysis, and in a very short time, healthcare professionals will be able to access to RutiRAM database to monitor and analyse ADRs occurring in their area of hospitalisation. In addition, in a new pharmacovigilance project recently implemented in our centre, RutiRAM data are also used to calculate the risk of inpatients to present an ADR during their stay. Other ongoing actions carried out in the hospital that can improve the drug and patient safety include educational interventions such as training and informing health professionals about ADRs, having experienced nurses for managing critical drugs in hospitalized patients, encouraging consultation with clinical pharmacologists for causality assessment of suspected ADR, monitoring plasma levels of drugs with narrow therapeutic margins, and using electronic prescription tools to prevent errors and drug-drug interactions.

The comparison of the number of ADRs between two periods was similar. When comparing the ATC group of involved drugs, the percentage of L group drugs increased in the second period, probably due to the increase of its use for the treatment of cancer or autoimmune diseases (Kesik-Brodacka, 2018). When comparing the SOC of reported ADRs, those corresponding to ‘Immune system disorders’ and ‘Skin and subcutaneous tissue disorders’ have increased, probably due to the incorporation of specialists in dermatology and allergology in the pharmacovigilance committee integrated in the HPVP, one of its main objectives is to increase ADR reporting. On the other hand, the ADRs corresponding to ‘Infections and infestations’ have decreased in the second period due to organizational circumstances that delayed the notification of 17 cases that occurred in that period and, consequently, were not included.

The exploratory data analysis found that medication errors were more frequent in hospital-acquired ADRs, which could be explained by the fact that most cases have been identified through the medication error committee; drug-drug interactions were more frequent in serious ADRs, which include infections related to immunosuppressants and bleeding related to antithrombotic agents (Marengoni et al., 2014; Létinier et al., 2021). Serious ADRs were related to elderly patients (Monteiro et al., 2021), while drugs ‘under additional monitoring’ were more commonly involved with younger patients. These age differences can be explained by the fact that older patients have comorbidities that can complicate ADRs and lead to serious outomes, while some of the drugs ‘under additional monitoring’ are for neoplastic diseases, which often affect adult patients.

### 4.1 Limitations and strengths

The study has several limitations. The primary limitation is associated with the inherent underreporting of ADRs in spontaneous reporting. This implies that these reported cases represent only a small fraction of the actual occurrences, estimated as 10% or less (Hazell and Shakir, 2006). It is necessary to recall that the incidence of ADRs cannot be obtained through spontaneous reporting because data on the number of patients exposed to a drug (denominator) and data on the number of patients with an ADR (numerator) are not known (Pal et al., 2013; Hazell and Shakir, 2006). Another limitation is the retrospective design of the study, which could affect the collection of some variables such as patients’ comorbidities or number of concomitant drugs, although it would not change the overall results. In addition, this study was conducted in a single centre where there is a specific HPVP with its own characteristics, which introduces a reporting bias that complicates the extrapolation of results and robust comparisons with pharmacovigilance programs in other hospitals. Some of the ADRs reported in the RutiRAM database are closely linked to the fact that clinical pharmacologists specialized in pharmacovigilance are members of clinical hospital committees such as the mortality committee, the committee for the prevention of medication errors and the committee for the prevention of infections in patients on immunosuppressive biologic drugs.

The study also has strengths. This is the first study conducted in Europe based on a HPVP with data on suspected ADRs for more than 10 years. This study includes ADRs across all areas related to the hospital where patients are admitted, receive treatments or surgery, home hospitalization and outpatient clinics, unlike most of the available studies, which only evaluate specific clinical areas or services within the hospital. Information collected from cases is very detailed, and its quality is high because the data were carefully collected and validated by clinical pharmacologists with expertise in pharmacovigilance (Begaud et al., 1994). We have assessed many variables not included in previous pharmacovigilance studies, such as drugs ‘under additional monitoring’, medication error, positive rechallenge, and ‘pharmacovigilance interest’. Some of the included drugs are used exclusively in hospitals. Furthermore, the study duration is sufficient to derive meaningful conclusions. An exploratory data analysis has also been conducted to identify associations between the study variables revealing new and interesting results.

## 5 Conclusions

This is the first study to provide an overview of ADRs reported in an HPVP over more than a decade. More than half of the ADRs were serious, which were related to older patients and drug-drug interactions. Almost two-thirds of the ADRs collected in the RutiRAM database are of sufficient quality to be classified as ‘pharmacovigilance interest’, and thus can contribute to signal detection and the issuing of drug alerts by pharmacovigilance systems. Analysing ADRs occurring in the hospital setting help to contribute to the improvement of patient safety with the implementation of specific actions to avoid medication errors or prevent ADRs, some of which can be generalized to other centres.

## Data Availability

The raw data supporting the conclusions of this article will be made available by the authors, without undue reservation.
